# Feasibility of RNA and DNA Extraction from Fresh Pipelle and Archival Endometrial Tissues for Use in Gene Expression and SNP Arrays

**DOI:** 10.1155/2013/576842

**Published:** 2013-10-26

**Authors:** Heather D. Kissel, Thomas G. Paulson, Karen Liu, Xiaohong Li, Elizabeth Swisher, Rochelle Garcia, Carissa A. Sanchez, Brian J. Reid, Susan D. Reed, Jennifer Anne Doherty

**Affiliations:** ^1^Division of Human Biology, Fred Hutchinson Cancer Research Center, Seattle, WA 98109, USA; ^2^Division of Vaccine and Infectious Disease, Fred Hutchinson Cancer Research Center, Seattle, WA 98109, USA; ^3^Department of Obstetrics and Gynecology, University of Washington School of Medicine, Seattle, WA 98105, USA; ^4^Department of Pathology, University of Washington Medical School, Seattle, WA 98105, USA; ^5^Division of Public Health Sciences, Fred Hutchinson Cancer Research Center, Seattle, WA 98109, USA; ^6^Department of Medicine, University of Washington, Seattle, WA 98105, USA; ^7^Department of Genome Sciences, University of Washington, Seattle, WA 98105, USA; ^8^The Geisel School of Medicine at Dartmouth, Hanover, NH 03755, USA; ^9^The Norris Cotton Cancer Center, Lebanon, NH 03756, USA

## Abstract

Identifying molecular markers of endometrial hyperplasia (neoplasia) progression is critical to cancer prevention. To assess RNA and DNA quantity and quality from routinely collected endometrial samples and evaluate the performance of RNA- and DNA-based arrays across endometrial tissue types, we collected fresh frozen (FF) Pipelle, FF curettage, and formalin-fixed paraffin-embedded (FFPE) hysterectomy specimens (benign indications) from eight women. Additionally, neoplastic and uninvolved tissues from 24 FFPE archival hysterectomy specimens with endometrial hyperplasias and carcinomas were assessed. RNA was extracted from 15 of 16 FF and 51 of 51 FFPE samples, with yields >1.2 **μ**g for 13/15 (87%) FF and 50/51 (98%) FFPE samples. Extracted RNA was of high quality; all samples performed successfully on the Illumina whole-genome cDNA-mediated annealing, selection, extension, and ligation (WG-DASL) array and performance did not vary by tissue type. While DNA quantity from FFPE samples was excellent, quality was not sufficient for successful performance on the Affymetrix SNP Array 6.0. In conclusion, FF Pipelle samples, which are minimally invasive, yielded excellent quantity and quality of RNA for gene expression arrays (similar to FF curettage) and should be considered for use in genomic studies. FFPE-derived DNA should be evaluated on new rapidly evolving sequencing platforms.

## 1. Introduction

Though endometrial carcinoma is the most common gynecologic malignant neoplasm [1], diagnostic capabilities and management of endometrial precancer (intraepithelial neoplasia) lag far behind those of cervical carcinoma [2]. A neoplastic continuum from simple, to complex, to atypical hyperplasia, to endometrial carcinoma is suggested from longitudinal epidemiologic studies [3–5]. Identification of molecular alterations present in various stages of endometrial neoplasia will provide the basis for early detection and therapeutics [6]. Present diagnostic capabilities utilizing histologic evaluation for endometrial hyperplasia/neoplasia alone are limited by poor diagnostic reproducibility [7] and relatively low prognostic value. Risk of progression to carcinoma among women with a diagnosis of endometrial hyperplasia with atypia is not well understood, though exposure to progestin therapy has been reported to be associated with an approximately 60% decreased risk of progression [4, 8]. Future studies that attempt to elucidate molecular biomarkers of endometrial hyperplasia progression risk will require the development of two methodologies: the ability to perform array studies from extremely small fresh endometrial samples, as well as high-fidelity large-scale affordable array interrogation of archived FFPE samples. Potential challenges exist with both. 

The growing field of genomic technologies has made large array-based studies of somatic mutations possible; whole exome sequencing and mutation detection have led to identification of potential driver cancer genes in endometrial cancer [9, 10]. Tissue archives of longitudinal endometrial neoplasia (intraepithelial neoplasm) specimens represent a potentially valuable resource for genomic studies, but they are typically comprised of formalin-fixed paraffin-embedded (FFPE) samples [4, 5]. High-quality nucleic acids necessary for gene expression profiling are most readily obtained from fresh or fresh frozen samples, as the purity and quantity of extracted RNA and DNA from archival tissues can be highly variable [11]. However, the whole-genome cDNA-mediated annealing, selection, extension, and ligation (WG-DASL) Assay (Illumina, San Diego, CA, USA) [12] has been implemented using partially degraded RNA extracted from FFPE tissues for breast carcinoma [13–15] and ovarian carcinoma [16], with varying degrees of success. It is essential to evaluate the quantity and quality of RNA and DNA obtained from endometrial FFPE samples and their performance on high-throughput arrays to determine the feasibility of using these readily available tissue archives to further our understanding of longitudinal progression of endometrial neoplasms and improve diagnostic accuracy and thereby, therapeutic choices.

Women diagnosed with endometrial neoplasia present with abnormal or heavy bleeding patterns and are usually first evaluated with office endometrial biopsies [17, 18], often sampled at multiple time points. Samples are obtained blindly using a disposable suction device placed into the uterus, which results in random sampling that is not oriented to uterine site. The most common device is a Pipelle. The small diameter (3 mm) and flexible plastic suction curette improve tolerability of this office procedure. Volumes of tissue samples obtained are usually 1-2 cubic centimeters (cc) or less than 1 cc in atrophic samples, and can be greater than 3 cc in proliferative samples, for example, endometrial hyperplasia/neoplasia. Moving forward, in the clinical scenario of a woman presenting with abnormal bleeding, we envision holding a small amount of fresh office endometrial biopsy specimen for biomarker analysis while retaining the bulk of the specimen for FFPE clinical diagnostic analysis. 

To address whether the quantity and quality of RNA obtained from office endometrial samples and archived FFPE endometrial tissues were suitable for downstream array analyses, we compared the quality and quantity of RNA isolated from three endometrial specimen types from the same individual (a) Pipelle suction (blind, random, small volume, single pass only); (b) curettage (nonblind, oriented fundus to cervix from bivalve hysterectomy specimen); and (c) FFPE surgical hysterectomy specimens, as well as paired endometrial hyperplasias and carcinomas and uninvolved myometrium from archived FFPE specimens. We also examined DNA yield and DNA performance on the Affymetrix SNP 6.0 platform in a subset of samples.

## 2. Materials and Methods

### 2.1. Study Participants and Biological Samples

This study was approved by the University of Washington Human Subjects Division. 

### 2.2. Fresh Tissue Collection

To assess whether quantity, quality, and performance of RNA and DNA obtained from different types of FF and FFPE endometrial samples from the same person varied by sample type, we enrolled eight women undergoing total abdominal hysterectomy for benign conditions in July and August of 2009. Indications for hysterectomy (not mutually exclusive) were irregular heavy bleeding (*n* = 6); uterine prolapse (*n* = 1), and fibroids (*n* = 4). For each patient, three types of samples were collected: (1) fresh Pipelle (CooperSurgical Inc.) biopsy; (2) fresh curettage biopsy; and (3) FFPE hysterectomy surgical sections. Immediately following removal of the uterus, a single pass with a Pipelle curette was performed and tissues were placed into tissue culture media (minimum essential media (MEM) with 5% fetal calf serum, 5 mM HEPES buffer, and 10% DMSO) on ice for storage at −30°C. No RNAlater sample was obtained as we had no data to guide whether the endometrial sample obtained with one pass would be of sufficient volume for both diagnostic and research purposes; our primary focus was on the performance of the MEM-preserved specimens. The uterus was then bivalved and the endometrium was sharply curetted in two passes from the fundus to the junction of the uterus and the cervix. One curettage sample was placed into MEM, and the second sample was placed into RNAlater (Qiagen, Valencia, CA, USA), a medium suitable for long-term storage of tissues for both RNA and DNA extractions. Both curettage samples were placed on ice at −30°C until frozen for storage at −70°C. The uterus was then sectioned and processed into FFPE hysterectomy samples for histopathology. Each method of tissue harvesting potentially samples different cell types. Pipelle biopsies sample the endometrial lining, while the curettage biopsies are comprised of predominantly endometrial tissue but may contain myometrial cells. The FFPE hysterectomy specimens contain both endometrial and myometrial cell types.

We were able to obtain sufficient tissue for analysis from 7 of 8 FF Pipelle (MEM), 8 of 8 FF curettage (MEM), 5 of 8 FF curettage (RNAlater) samples, and all 8 hysterectomy samples (FFPE) (Table 1(a)).

### 2.3. Archival Tissue Collection

To assess quantity and quality of DNA and RNA from older archived clinical samples and performance on the WG-DASL and SNP 6.0 platforms, we selected 24 FFPE hysterectomy specimens: 6 complex hyperplasias; 6 atypical hyperplasias; and 12 carcinomas from the University of Washington Pathology Department. The samples were collected between 1999 and 2009 and stored for an average of 4.4 years (range 1 to 11 years) before tissue cores were collected for RNA and DNA extraction.

All FFPE surgical histopathology slides were reviewed by a pathologist to identify and mark blocks with representative and adequate areas of the various tissue types for RNA and DNA extraction. For the specimens with hyperplasia, uninvolved endometrium was marked for sampling 7–10 millimeters away from the involved hyperplastic endometrium. For the specimens with carcinoma, uninvolved myometrium was identified for sampling from separate blocks without carcinoma. Samples were obtained from the paraffin blocks by coring the marked area(s) of the identified tissue with a warm 18-gauge needle. Approximately 15 cores were collected per sample and the total core weight was recorded; up to 37 mg of paraffin-embedded tissue (range, 11–37 mg). Cores collected from each tissue type were placed into separate microcentrifuge tubes, deparaffinized, and extracted using the Qiagen Blood and Tissue DNeasy spin columns according to the manufacturer's protocol.

In total, we obtained FFPE tissue for 51 samples, including the benign hysterectomy samples described above (*n* = 8; Table 1(a)), as well as complex hyperplasia (*n* = 6 involved, *n* = 6 uninvolved); atypical hyperplasia (*n* = 6 involved, *n* = 6 uninvolved; Table 1(b)); and endometrial carcinoma (*n* = 12 involved, *n* = 7 uninvolved myometrium; Table 1(c)). We were unable to obtain uninvolved myometrial samples from 5 of the 12 endometrial carcinoma samples because there were no tissue blocks without carcinoma for these individuals.

### 2.4. RNA Extraction, Expression Array, and RT-PCR Validation of Expression Array

RNA extraction was performed on samples with sufficient tissue. For the FF samples, the specimens were split for RNA and DNA extraction, and for FFPE tissues, the weight of the cores for RNA extraction was recorded (Table 1(a)). Extractions were performed using the RecoverAll Total Nucleic Acid Isolation Kit (cat no. AM1975, Applied Biosystems/Ambion, Austin, TX, USA). RNA was quantitated using the NanoDrop ND-1000 spectrophotometer. Quality was assessed by measuring the Ct threshold using QuantiTect SYBR-Green RT-PCR mix using the protocol with the WG-DASL kit (Illumina, San Diego, CA, USA) on a quantitative real-time PCR (qRT-PCR) system (Applied Biosystems/Life Technologies 7900HT, Carlsbad, CA) in the Fred Hutchinson Cancer Research Center (FHCRC) Genomics Core.

The amount of input RNA recommended by Illumina for the WG-DASL (Illumina, San Diego, CA, USA) is 10–100 ng for RNA derived from FF samples and 50–200 ng for RNA derived from FFPE samples. Therefore, samples were excluded if they yielded less than 40 ng of RNA. Thirteen FF and 49 FFPE samples were assayed. To test reproducibility of the assay, we randomly selected 8 FF samples (4 Pipelle; 4 curettage) and 26 FFPE samples (4 benign; 3 complex, both involved and uninvolved; 3 atypical involved and uninvolved; and 5 carcinoma, both involved and uninvolved) to run in replicate for a total of 21 FF and 75 FFPE samples. The samples were processed and run on the WG-DASL arrays by the FHCRC Genomics Shared Resource according to the manufacturer's protocol. We examined whether the quality and quantity of the RNA varied by tissue type and fixation method, and for the FFPE samples, whether RNA quality varied by year of sample processing. Various performance metrics were examined, including the average number of probes detected (out of the 24,526 transcripts assayed), the average number of genes detected (out of the 18,391 genes assayed), the average signal p95, which is the 95th percentile of the probe intensities on the array, and the signal-to-noise ratio, which compares the strength of the signal to the background signal. Using Genome Studio (Illumina, San Diego, CA, USA), a detection *P* value was calculated for each transcript, which represents the probability of observing a given transcript if in fact the signal is not above the noise, with the background defined using negative control probes. We examined two levels of confidence: *P* < 0.01 and *P* < 0.05. While a larger number of probes are defined as detected for *P* < 0.05, they might not be as reproducible as the probe set defined by *P* < 0.01. Probe concordance is defined as the percentage of the number of probes with matching detected calls (at *P* < 0.01 or *P* < 0.05) in two replicate samples, over the total number of probes detected in either of the two samples.

To validate the relative expression values obtained by WG-DASL, expression levels of six genes were examined by qRT-PCR in a subset of RNA samples (*n* = 44). Five genes (*PTEN, CD79B, CD82, S100A4, *and* FOLR1*) were selected because they have been reported to be associated with endometrial hyperplasia or endometrial carcinoma (http://www.proteinatlas.org/) [6, 19], and one (*KCNMA1*) was selected because we observed a wide range of expression levels (as assayed using WG-DASL) across the tissues examined. The 44 samples included 13 FF (7 MEM Pipelle and 6 MEM curettage) and 31 FFPE (8 benign hysterectomy, involved tissue from 6 complex hyperplasias, 5 atypical hyperplasias, and 12 endometrial cancers). All 44 samples were run in duplicate, resulting in 88 samples for a total of 528 data points. Averaged ratios of expression obtained by WG-DASL and qRT-PCR were compared for the six genes. We determined the fold difference in expression of a given gene between two sets of tissues by dividing the level of expression in one tissue by the level in the other. We then compared the fold differences between the tissue types for measures obtained by DASL versus those obtained by qRT-PCR. The two different fold changes for a given gene were plotted as *X* and *Y* coordinates on a correlation graph and an *R* ^2^ value was calculated to determine how well the two sets of measures were correlated across all 6 genes.

### 2.5. DNA Extraction and Genotyping

For FF samples, DNA extraction was performed on 20 Pipelle and curettage samples stored either in MEM (7 Pipelle and 8 curettage samples; one Pipelle did not have sufficient tissue for extraction) or RNAlater (5 of 8 curettage samples had sufficient tissue; no Pipelle samples were stored in RNAlater as the entire Pipelle samples for research were placed in MEM to maximize quantity). FF samples were minced with scalpel blades and sheared with NST buffer (146 mM NaCl, 10 mM Tris Base (pH 7.5), 1 mM CaCl_2_, 0.5 mM MgSO_4_, 0.05% Bovine serum albumin (BSA), 21 mM MgCl_2_, and 0.2% Igepal) with a 1 cc syringe as needed prior to extraction using Puregene DNA Isolation Kit (Gentra Systems, Inc., Minneapolis, MN, USA). DNA was quantitated with PicoGreen (Quant-iT dsDNA Assay, Invitrogen, Carlsbad CA, USA).

For FFPE samples, we performed DNA extraction on 12 endometrial cancers with paired uninvolved myometrium (*n* = 7; 5 cancers did not have corresponding blocks of uninvolved tissue). The samples were deparaffinized and extracted using the Blood and Tissue DNeasy spin columns according to the manufacturer's protocol (Qiagen, Valencia, CA, USA). DNA quality was assessed by measuring its size and concentration using a microfluidics-based platform offering qualitative and semiquantitative analysis (Agilent 2100 Bioanalyzer) with the DNA 7500 LabChip assay (Agilent Technologies, Santa Clara, CA, USA) in the Fred Hutchinson Cancer Research Center (FHCRC) Genomics Core.

To evaluate the performance of DNA derived from RNAlater and FFPE, we planned to genotype the DNA samples from the 5 FF curettage samples preserved in RNAlater and the 7 paired FFPE carcinomas and myometrium from uninvolved tissue blocks on the Genome-Wide Human SNP Array 6.0 (Affymetrix, Santa Clara, CA, USA) according to the standard Affymetrix protocol by HudsonAlpha Institute for Biotechnology, Huntsville, AL, USA. According to that protocol, DNA was digested with Nsp I and Sty I restriction enzymes and ligated to adaptors to amplify DNA prior to genotyping. However, the DNA from FFPE samples was fragmented and failed to amplify. Therefore, these samples were not genotyped. DNA from the RNAlater samples was resuspended at 50 ng/*μ*L prior to analysis on arrays.

## 3. Results

### 3.1. RNA Quantity and Quality and WG-DASL Array Performance and Reproducibility

We extracted RNA from 15 of 16 FF MEM-preserved biopsy samples (7 Pipelle, 8 curettage) and 51 of 51 FFPE tissues. The extraction was considered successful if the sample yielded ≥1.2 *μ*g of RNA and had a 260/280 ratio of 1.80 to 2.20. Extraction was successful for 13/15 (87%) FF and 50/51 (98%) FFPE samples. All three failures were attributed to low yields <1.2 *μ*g (2 curettage samples and one uninvolved FFPE sample from complex hyperplasia). One postmenopausal individual (patient ID no. 3) was found to have an atrophic endometrium. Not surprisingly, there was insufficient tissue from both Pipelle and curettage (MEM and RNALater) for analysis (Table 1(a)). The yields from the FF tissues were higher than those from the FFPE tissues, but the extracted RNA was very pure for all of the sample types, as evidenced by the 260/280 ratios being close to 2.0, which is the 260/280 ratio for pure RNA (Table 2). 

A prequalification qRT-PCR assay was performed to test the quality of the RNA for optimal performance prior to running the WG-DASL array. A Ct threshold above 29 cycles was used as a cutoff to indicate poor quality samples. While the FF samples performed slightly better as evidenced by lower numbers of Ct cycles than did the FFPE samples, all 63 extracted samples passed the prequalification assay (Table 2).

The overall performance of the RNA samples on the WG-DASL array was excellent and was only slightly better for the FF samples than the FFPE samples with respect to the 95th intensity percentile (p95) and signal-to-noise ratio. The average number of probes detected (*P* < 0.05) was similar for both FF and FFPE tissues, with 75% (18421/24526) detected for FF, 73% (17959/24526) detected for FFPE, and 69% of genes detected for both groups (12780/18401 for FF and 12296/18401 for FFPE). The average number of probes detected at *P* < 0.05 and *P* < 0.01, respectively, ranged from 17,197 (70.1%) and 15,640 (63.8%) for benign hysterectomy FFPE tissue to 18,640 (76.0%) and 17,064 (69.6%) for FF Pipelle tissue. The proportion of genes detected was lowest for both benign hysterectomy FFPE tissue and FFPE carcinoma at 64.8%, and highest for Pipelle, at 70.2% (Table 2). The Pipelle and curettage samples were highly comparable with an average expression level correlation *R* ^2^ = 0.939. The average expression level correlations for FF Pipelle and FF curettage compared to their matched FFPE hysterectomy samples were similar (0.690 and 0.692, resp.). 

To assess the reproducibility of the WG-DASL array, we assessed the probe correlation for sample replicates. Expression levels were highly correlated for both the FF and FFPE replicate samples (*R* ^2^ = 0.991 and *R* ^2^ = 0.985, resp.). Probe detection concordance rates of the replicates were calculated at *P* values of <0.05 and <0.01, and all samples had high concordance with an average of 96% gene overlap at both *P* values (Table 2). The lowest concordance rates were observed in the carcinomas at 94%. There was no difference in the quality of FFPE RNA samples, probe concordance across replicates, RNA quality (260/280 ratio), or quantity (RNA yield), by year of sample collection (data not shown).

### 3.2. Validation of WG-DASL Array with qRT-PCR

Expression of six genes was assayed using qRT-PCR on the same RNA samples used for the WG-DASL arrays. Correlations between fold differences in expression assayed using WG-DASL and qRT-PCR were evaluated for each gene between tissue types (FF Pipelle and curettage, FFPE atypical and complex hyperplasia, FFPE cancer, and uninvolved myometrium). Agreement between the two methods was good for Pipelle and curettage (*R* ^2^ = 0.90) and cancer and myometrium (*R* ^2^ = 0.82) but was poor for atypical and complex hyperplasia samples (*R* ^2^ = 0.02). To investigate whether the atypical or complex hyperplasia RNA samples had deteriorated over time, we examined the fold change in expression of the panel of genes as determined by WG-DASL and qRT-PCR in the atypical and complex hyperplasia samples compared to FFPE hysterectomy from the same women. The gene expression changes between atypical hyperplasia and FFPE hysterectomy assayed using the WG-DASL and qRT-PCR were well correlated (*R* ^2^ = 0.82), but this was not the case for complex hyperplasia compared to FFPE hysterectomy (*R* ^2^ = 0.18). We did not find evidence of suboptimal qRT-PCR due to technical issues (e.g., well position in the PCR block), suggesting the discrepancy was due to sample degradation specific to the complex hyperplasia samples in the time between the WG-DASL and qRT-PCR analyses (36 weeks).

### 3.3. DNA Quantity, Quality, and Genotyping Success

DNA extraction was successfully performed on all 20 FF samples and all 19 FFPE samples (12 carcinomas and 7 uninvolved myometrium) with excellent yields from all sample types. The average DNA yield was considerably higher for MEM Pipelle (46.4 *μ*g) than MEM curettage (27.3 *μ*g; Table 1(a)), though the DNA yield from RNAlater curettage was the highest (54.1 *μ*g). The DNA yield from FFPE samples was more than adequate for genomic studies (40.2 *μ*g). Genotyping was planned only for the 5 RNAlater-stored curettage samples and 14 of the FFPE samples (7 carcinomas and their paired uninvolved myometrium samples). However, because DNA fragmentation of the FFPE samples was detected, these samples were not genotyped. Of the five FF RNAlater-stored curettage samples, one failed due to low input DNA. The remaining four samples performed well, with call rates of 98.2% to 99.3% (mean, 98.8%). 

## 4. Discussion

This pilot study assessed the feasibility of using fresh and FFPE uninvolved, hyperplastic, and endometrial carcinoma specimens collected during routine clinical care for high-throughput, array-based, and quantitative methodologies. We demonstrated the ability to extract high-quantity and-quality RNA from both FF and FFPE specimens for all tissue types and showed generally successful performance on the WG-DASL platform. We also demonstrated that DNA derived from fresh samples stored in RNAlater can be used successfully for genotyping on the Affymetrix SNP 6.0 platform. The use of RNAlater has implications for simplifying clinical tissue collection, since samples can remain at room temperature during surgery rather than stored on ice as is necessary for samples placed in MEM. The SNP 6.0 array platform was not successful for DNA isolated from FFPE carcinomas and myometrium, despite there being a substantial volume of DNA. It is possible that our FFPE-derived DNA sample preparation protocol was not optimal; others have had success with the SNP 6.0 platform on DNA derived from FFPE tissue after implementing adjusted preparation protocols to improve hybridization performance and a modified data analysis procedure [20]. The protocol we used to collect DNA from FFPE tissues yielded far more DNA than is necessary for most genomic applications; future studies could take a smaller number of cores for DNA extraction to preserve the tissue for additional assays. The techniques validated in this pilot study have immediate potential to be used in samples from our existing longitudinal, retrospective cohort [4, 8] and to ultimately be translated to future clinical applications with specimens collected during outpatient gynecologic visits for abnormal bleeding in women at risk for endometrial neoplasia.

Of particular interest is the assessment of the quality and quantity of RNA extracted from Pipelle specimens, since this method of endometrial sampling is minimally invasive and can be performed repeatedly over time. We demonstrated that single pass Pipelle tissue specimens (disordered blinded specimens) are equivalent to curettage specimens with regard to DNA and RNA yield and RNA performance on the WG-DASL array. A limitation of our study was a lack of direct comparison of WG-DASL results between FF Pipelle and FFPE Pipelle samples. To our knowledge, RNA and DNA quality and quantity from Pipelle samples have not been evaluated previously. Because we needed to assure sufficient endometrium for clinical diagnostic procedures, and the amount of tissue needed to extract high-quality RNA and DNA was unknown, we conservatively chose to use the remaining sample for FF analysis. Given that the optimal Pipelle endometrial sampling process typically includes multiple (rather than single) uterine passes, and that we observed high RNA and DNA yields from the single pass Pipelle specimens, we propose that tissue collected in each pass could be split to use for diagnostic histopathologic evaluation and for research purposes.

We observed moderate success with RNA expression assayed using the WG-DASL method. While some prior studies have reported concordance between FF and FFPE samples [13], others have described poor correlations at the gene or probe level [15, 16], noting that combinations of genes (particularly those already identified as members of a predictive signature, that is, for ovarian cancer subtypes based on The Cancer Genome Atlas data) performed reasonably well. Given that there are no gene expression signatures to date that identify endometrial hyperplasia subgroups that are likely to respond to progestin, or are likely to progress, it is of critical importance to have complete and reliable gene expression data. Thus, while the WG-DASL approach appears to work reasonably well for validation, it is not as effective for discovery and is therefore not the ideal platform to use to identify such subgroups. 

Sequencing of both RNA and DNA derived from FFPE tissues is feasible, but performance of these methods will need to be assessed within specific endometrial sample types [21, 22]. Development of novel analytic methods for quality control of RNA expression data generated from FFPE tissues, such as the quality control pipeline described by Waldron et al. [23], are particularly important as data from multiple study sites over longer time periods are combined. Additionally, as new methods are developed, such as a novel method to simultaneously extract DNA, RNA, and microRNA from a single FFPE sample without splitting it [24], performance of the methods will need to be evaluated for endometrial samples.

## 5. Conclusions

Our study demonstrates that the Pipelle specimen, which is a common preferred method of clinical endometrial sampling in women with abnormal bleeding, can be used effectively for RNA expression studies and also provides a high yield of DNA. We propose that in the future, while most of the Pipelle would be reserved for FFPE diagnostic assays, a portion of the sample could be retained for fresh tissue analysis. Until endometrial biomarker studies become a routine aspect of care, our study demonstrates that instituting research practices that collect fresh specimens as well as retaining the majority of the specimen for FFPE should not affect current diagnostic capabilities and health outcomes. RNA expression data from FF Pipelle samples can be used to generate prognostic signatures, which can then be validated in archived FFPE tissues. Interrogation of longitudinal endometrial FFPE tissue banks to better understand alterations that occur in the progression of endometrial neoplasms is certainly feasible.

## Figures and Tables

**Table:** (a)

Study ID	Specimen collection date	Age	Menopause status		Pipelle, MEM				Curettage, MEM				Curettage, RNAlater		Hysterectomy, FFPE	
					DNA yield (*μ*g)	Weight of RNA tissue (mg)	RNA yield (*μ*g)		DNA yield (*μ*g)	Weight of RNA tissue (mg)	RNA yield (*μ*g)		DNA yield (*μ*g)		Weight of RNA tissue (mg)	RNA yield (*μ*g)
1	2009	46	Pre		24	31	62		26.6	26	50.1		35		20	13.4
2	2009	50	Peri		n/a*	29	23		8.5	29	32.8		28.1		21	16.8
3	2009	70	Post		101.9	n/a*	n/a*		23.4	n/a^§^	0^†^		X^‡^		21	7.8
4	2009	45	Pre		20.8	19	25.4		40.3	30	11.8		21		24	18.2
5	2009	51	Pre		37.7	31	20.9		17.5	n/a^§^	0.5^†^		X^‡^		25	2.7
6	2009	52	Peri		69	32	27.9		35.8	18	25.1		X^‡^		21	3.2
7	2009	47	Peri		25.3	27	44		48.7	20	34		49		21	4.4
8	2009	48	Pre		46.3	23	44.6		17.2	26	42.2		137.5		37	26
			Average yield		*46.4 *	*27.4 *	*35.4 *		*27.3 *	*24.8 *	*32.7 *		*54.1 *		*23.8 *	*11.6 *

*Not enough tissue remaining for nucleic acid extraction.

^†^Not enough yield to use tissue on downstream applications; <1.2 *μ*g. These are not included in the average yield.

^‡^No tissue available.

^§^Too little tissue to weigh accurately.

**Table:** (b)


Complex hyperplasia, *n* = 6									
Study ID	Specimen collection date	Age	Menopause status		Involved			Uninvolved	
					Weight of RNA tissue (mg)	RNA yield (*μ*g)		Weight of RNA tissue (mg)	RNA yield (*μ*g)
									
9	2000	65	Post		16	12.6		11	24.9
10	2001	53	Post		23	16.1		28	18.3
11	2003	38	Pre		27	20.9		27	0.2^†^
12	2005	38	Pre		36	1.6		32	14
13	2008	55	Post		24	48.5		25	19.1
14	2008	62	Post		25	3.1		17	5.7
			Average yield		*25.2 *	*17.1 *		*23.3 *	*16.4 *
15	2001	32	Pre		21	10.8		28	18.6
16	2003	54	Pre		29	8.2		27	18.2
17	2003	69	Post		24	17.8		26	12.1
18	2005	43	Peri		27	9.9		26	9.7
19	2005	54	Pre/Peri		25	8		25	6.9
20	2008	72	Post		29	11.8		26	21.4
			Average yield		*25.8 *	*11.1 *		*26.3 *	*14.5 *

**Table:** (c)

		Endometrial carcinoma, *n* = 12									
Study ID	Specimen collection date	Age	Menopause status		Involved				Uninvolved		
					DNA yield (*μ*g)	Weight of RNA tissue (mg)	RNA yield (*μ*g)		DNA yield (*μ*g)	Weight of RNA tissue (mg)	RNA yield (ng/ul)
21	1999	70	Post		14.7	16	30		40.2	23	43.1
22	1999	84	Post		89.7	32	36.4		18.3	24	6.8
23	1999	80	Post		8.7	26	20.9		36.8	28	13.7
24	2004	77	Post		12.5	25	29.6		51.3	29	6.1
25	2004	70	Post		33.2	30	14.7		57.3	34	11
26	2004	55	Post		83.04	21	27.4		X^‡^	X^‡^	X^‡^
27	2007	81	Post		40.9	23	19.4		X^‡^	X^‡^	X^‡^
28	2007	59	Post		17.38	35	27.1		39.4	28	6.4
29	2007	63	Post		9.6	31	11.1		X^‡^	X^‡^	X^‡^
30	2009	56	Peri		81.5	24	32.1		35.2	24	10.1
31	2009	44	Pre		49.2	32	18.1		X^‡^	X^‡^	X^‡^
32	2009	52	Post		44.6	32	39.7		X^‡^	X^‡^	X^‡^
			Average yield		*40.4 *	*27.3 *	*25.5 *		*39.8 *	*27.1 *	*13.9 *

*Not enough tissue remaining for nucleic acid extraction.

^†^Not enough yield to use tissue on downstream applications; <1.2 *μ*g. These are not included in the average yield.

^‡^No tissue available.

^§^Too little tissue to weigh accurately.

**Table 2:** RNA quality metrics and assay performance.

	Benign				Complex hyperplasia			Hyperplasia with atypia			Cancer	
	FF Pipelle	FF Curettage	FFPE		Normal	Involved		Normal	Involved		Normal	Involved
RNA Extraction												
Number successfully extracted (over number attempted)*	7/7	6/8	8/8		5/6	6/6		6/6	6/6		7/7	12/12
Mean RNA yield^†^ (*μ*g) (range)	35.4 (20.9–62.0)	32.7 (25.1–50.4)	11.6 (2.7–26)		16.4 (5.7–24.9)	17.1 (1.6–48.5)		14.5 (6.9–21.4)	11.1 (8.0–17.8)		13.9 (6.1–43.1)	25.5 (11.1–39.7)
Mean 260/280 ratio^†^ (range)	2.05 (2.01–2.08)	2.06 (2.05–2.09)	2.00 (1.9–2.0)		2.02 (1.93–2.05)	2.05 (1.91–2.1)		2.02 (1.97–2.05)	1.98 (1.8–2.03)		2.04 (1.93–2.09)	2.00 (1.9–2.1)
DASL Pre-qualification^‡^												
Mean Ct threshold (range)	15.7 (15.0–16.4)	15.7 (15–18.7)	21.0 (18.0–25.4)		20.9 (19.6–22.5)	19.5 (18.2–20.7)		20.5 (19.3–21.3)	19.9 (20.0–21.7)		21.5 (19–24.7)	21.0 (18.5–27.6)
DASL Expression Array												
Array pass rat*e* ^§^	7/7	6/6	8/8		5/5	5/5		6/6	6/6		7/7	12/12
Average number of probes detected (of 24,526, *P* < 0.05)	18,640 (76.0%)	18,165 (74.1%)	17,197 (70.1%)		18,455 (75.2%)	18,244 (74.4%)		18,239 (74.4%)	18,446 (75.2%)		18,177 (74.1%)	17,648 (72%)
Average number of probes detected (of 24,526, *P* < 0.01)	17,064 (69.6%)	16,637 (67.8%)	15,640 (63.8%)		16,912 (69.0%)	16,846 (68.7%)		16,647 (67.9%)	16,823 (68.6%)		16,442 (67.0%)	15,874 (64.7%)
Average number of genes detected (of 18,401, *P* < 0.01)^¶^	12,917 (70.2%)	12,630 (68.6%)	11,931 (64.8%)		12,434 (67.6%)	12,580 (68.4%)		12,642 (68.7%)	12,645 (68.7%)		12,348 (67.1%)	11,930 (64.8%)
Average Signal p95^¶^	7,436	8,495	6,630		6,906	6,944		7,202	6,921		6,703	6,725
Signal-to-noise ratio^¶^	191	188	184		179	184		176	179		178	192
Replicate Concordance												
Number of replicates run on array	3	4	4		3	3		3	3		5	5
Correlation of expression levels (*r* ^2^)	0.99	0.99	0.99		0.99	0.99		0.98	0.99		0.99	0.98
Probe concordance, *P* < 0.05	0.97	0.98	0.96		0.96	0.96		0.95	0.96		0.96	0.94
Probe concordance, *P* < 0.01	0.98	0.98	0.97		0.96	0.97		0.96	0.96		0.96	0.94

*Extractions were defined as successful if the yield was >1.2 *μ*g and the 260/280 ratio was between 1.80 and 2.20, as pure RNA has a 260/280 ratio of 2.0.

^†^Excluding the three samples that had yields <1.2 *μ*g.

^‡^A Ct threshold of 29 or below qualifies RNA for the DASL array.

^§^All replicates also passed.

^¶^In our high-throughput facility, the average number of genes detected is 10,000, the average p95 signal is 5,000, and the average signal-to-noise ratio is 40.
